# Sensor Data Reconstruction for Dynamic Responses of Structures Using External Feedback of Recurrent Neural Network

**DOI:** 10.3390/s23052737

**Published:** 2023-03-02

**Authors:** Yoon-Soo Shin, Junhee Kim

**Affiliations:** Department of Architectural Engineering, Dankook University, Yongin 16890, Republic of Korea

**Keywords:** structural health monitoring, sensor data reconstruction, machine learning, recurrent neural network, external feedback

## Abstract

An event of sensor faults in sensor networks deployed in structures might result in the degradation of the structural health monitoring system and lead to difficulties in structural condition assessment. Reconstruction techniques of the data for missing sensor channels were widely adopted to restore a dataset from all sensor channels. In this study, a recurrent neural network (RNN) model combined with external feedback is proposed to enhance the accuracy and effectiveness of sensor data reconstruction for measuring the dynamic responses of structures. The model utilizes spatial correlation rather than spatiotemporal correlation by explicitly feeding the previously reconstructed time series of defective sensor channels back to the input dataset. Because of the nature of spatial correlation, the proposed method generates robust and precise results regardless of the hyperparameters set in the RNN model. To verify the performance of the proposed method, simple RNN, long short-term memory, and gated recurrent unit models were trained using the acceleration datasets obtained from laboratory-scaled three- and six-story shear building frames.

## 1. Introduction

Structural health monitoring (SHM) systems typically include sensors and data acquisition systems and are, thus, referred to as sensor-based monitoring systems. Because monitoring systems increasingly rely on sensor technology, effective management of sensor networks has become important [1,2]. However, sensor faults are frequent and inevitable in real systems owing to problems, such as noise, degradation, and harsh environmental conditions [3,4]. In particular, the sensors installed in buildings are constituted of various elements, such as structures, facilities, and exterior materials, that are difficult to maintain and repair [5,6]. Data loss from the failed sensor channels, which results in sparse sensor networks [7], significantly degrades the structural condition assessment and causes errors in the assessment of the structural health status [8,9]. Data recovery of defective sensor channels is crucial not only for operating sensor networks but also for managing the quality of the SHM [10,11].

Over the last decade, the recovery of missing data caused by the failure of parts of the sensor network deployed in structures has been extensively studied [12,13,14]. If sensors belong to dense sensor networks deployed in a structure, each sensor shares a certain level of correlation with the others [15]. The reconstruction of defective sensor data utilizes this correlation by analyzing the data measured by different sensors [16]. The correlation between sensor data collected for the dynamic behaviors of structures consists of a spatial correlation between sensor channels at a time instant and a temporal correlation in a sequence of the timeline, which is often referred to as spatiotemporal correlation [17]. Correlation analysis for sensor data reconstruction has been mainly conducted using the numerical method of black box model analysis instead of theoretical dynamic analysis based on mechanical interpretation.

Data reconstruction techniques using artificial neural networks have shown excellent performance in recovering lost data by learning nonlinear patterns in correlated data. For example, a convolutional neural network (CNN) was adopted to reconstruct the acceleration responses of bridges indirectly by processing transformed images of time-series data [18,19]. Because the transformation of time series to images involves an inherent loss of partial information in the time domain, recurrent neural networks (RNNs) have been applied in the field of data reconstruction as an alternative to CNN. The RNN is a looped architecture specialized for learning temporal patterns in time-sequential data and is, therefore, utilized in research on natural language and speech processing [20,21,22]. A dominant feature of an RNN is its cyclic structure, in which the current neural networks share information from previous neural networks at the learning stage. The cyclic structure is effective for processing data from a dynamic system in which the current state affects the state in subsequent time steps [23,24]. A series of variants of the RNN, such as long short-term memory (LSTM) [25], gated recurrent unit (GRU) [26], bidirectional RNN (BRNN) [27], and bidirectional LSTM (Bi-LSTM) [28], were also used to reconstruct lost data, and their superior performance was reported [17,29,30].

Numerous hyperparameters determining the network structures, such as the number of input vectors and hidden layers, learning rate, cost function, regularization parameter, batch size, and training epoch, must be set before training the RNN models [31,32]. The repetitive trials to fix them and the subsequent evaluation outputs of the models are referred to as hyperparameter tuning [33]. The number of input vectors and hidden layers directly affects the number of parameters in the model and accordingly determines the accuracy, computational amount, and prediction speed of the model. Setting optimized hyperparameters for excellent data reconstruction performance is a cumbersome task, and the related process is often omitted from data reconstruction studies [29,34].

In this study, by leveraging the spatial correlation between sensor channel data more than the temporal correlation on the dynamic behavior of structures, an RNN model with external feedback is proposed for the reconstruction of data. The proposed method generates a robust and precise model by explicitly feeding back the reconstructed data from the RNN model to the input dataset for the next time step. Accordingly, the advantage of improving the accuracy of the recovered data was confirmed by simplifying the hyperparameter tuning process of the RNN model to recover the lost data. The remainder of this paper is organized as follows: Section 2 describes the architecture of a general RNN model to apply and verify the proposed technique. Section 3 explains the RNN model training and data recovery method that employs the proposed technique. In Section 4, a performance verification of the proposed method is conducted by utilizing the vibration data collected from the structural model. The paper concludes with a brief summary and discussion in Section 5.

## 2. RNN Architecture for Sensor Data Reconstruction

**Figure 1:**
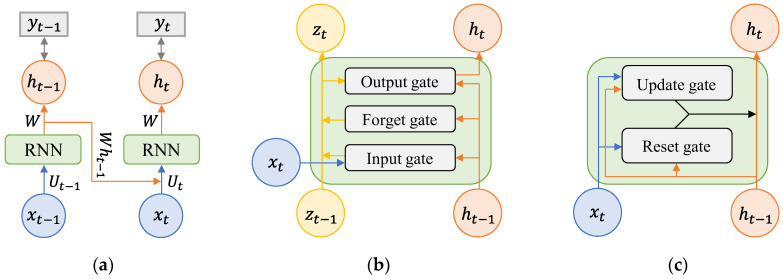
The RNN and its variant models. (**a**) Simple RNN. (**b**) LSTM. (**c**) GRU.

Various RNN models have been derived according to parameter structures, such as the type of input–output data and the weight inside the layer [28,35]. In the training time series, the RNN structure was upgraded so that parameters can contain time-series characteristics for a longer period of time. Representative RNN models, simple RNN, LSTM, and GRU are presented in Figure 1. These are used as reference models for performance comparison of the difference in layer structures in lost data reconstruction studies [36,37].

A simple RNN, which is a chain of general neural networks, sequentially delivers input data to the cells of adjacent layers, that is, it is the basic form of a recurrent model. Because a cell that has delivered data does not store information about the transmitted data, it cannot remember long-term time-series information. Figure 1a shows the structure of a simple RNN with one memory between the input and output networks. When the activation function is used as a hyperbolic tangent, the memory $h_{t}$ used to predict the output vector is defined as

(1) $$h_{t}=\tan h\left(U_{t}x_{t}+Wh_{t-1}+b\right)$$

where x is an input vector, U and W are flexible matrices, and b is the bias vector. The training of the model is a process of adjusting the weights such that $x_{t}$ approaches the correct value $y_{t}$ for predicting $h_{t}$ generated by $U_{t}$, W and $h_{t-1}$. Because $h_{t-1}$ of the previous time step is involved in predicting $h_{t}$ of the current time step, a recurrent structure of the neural network is established. This is a structure in which the results of the previous step are fed back to the computation of the current step, and through this, the RNN enables the processing of time-series data. 

LSTM and GRU are models in which the cell structure that connects the input and output vectors is modified to improve the low long-term dependency of a simple RNN (Figure 1b,c). The biggest feature of LSTM is that the cell state parameter, $z_{t}$, is additionally shared between adjacent layers, and the value of the existing input vector is preserved so that the long-term memory storage capacity is improved. Several parameters determined by the size of the input and output vectors exist inside a single layer, and the output gate, forget gate, and input gate composed of these parameters protect and control the cell state. In particular, the forget gate is built in to directly determine how much to remember and how much to forget the value of the previous vector. GRU is a simple type of LSTM in which the structure of the LSTM is modified. GRU has fewer parameters and faster training than LSTM because the forget gate and input gate of LSTM are merged into a single layer called the update gate.

## 3. Internal and External Feedback in RNN Model for Sensor Data Reconstruction

**Figure 2:**
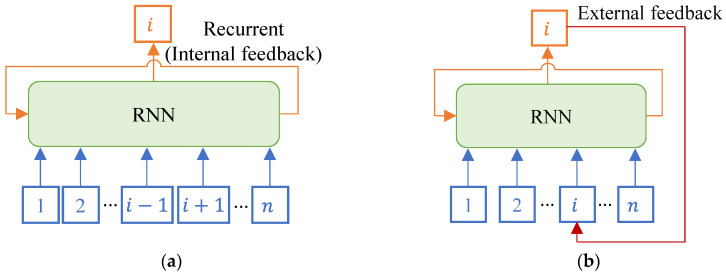
Schematic diagram of internal and external feedback in the RNN model. (**a**) Internal feedback. (**b**) External feedback.

Figure 2a depicts a conventional training RNN model for the reconstruction of the i-th lost channel in n sensor networks. As shown in the figure, an input dataset consisting of n−1 channels excluding the i-th channel was used for training the RNN model. Sensor data reconstruction utilizes the correlation in sensor data in black-box analysis. With black-box analysis of spatial correlation in data from sensor channels and temporal correlation in a sequence of time series, the RNN model generates data from the lost channel. In particular, the temporal correlation is analyzed using the inherent recurrence in the RNN model that utilizes the output of the previous step at every time step. In this study, inherent recurrence is referred to as internal feedback.

In addition to internal feedback, a method of feeding the reconstructed loss channel data back to the input dataset is proposed in Figure 2b. In the reconstruction of the i-th lost channel data in the n sensor network, the output of the i-th channel reconstructed from the model is composed of the input dataset in the next step. Thus, the input dataset of the RNN model consists of n datasets. In this study, the looped structure is referred to as the external feedback.

**Figure 3:**
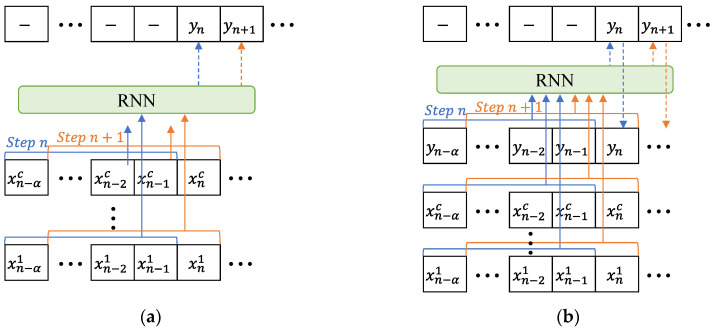
Input and output data flow of internal and external feedback in the RNN. (**a**) Internal feedback. (**b**) External feedback.

Figure 3 shows a schematic diagram representing the input–output dataset relationships of the RNN model for both the conventional and external feedback methods. As shown in Figure 3a, the input and output, that is, the reconstruction channels, are independently set to $X_{t}^{c}$ and $y_{t}$, respectively, where c and t are the channel number and time step of the operating sensors, respectively. The input dataset $X_{n}^{c}$ for predicting the n-th element $y_{n}$ of the $y_{t}$ channel consists of the following matrix of size (c×α).

(2) $$X_{n}^{c}=\left[\begin{array}{ccc} x_{n-\alpha }^{c} & \cdots & x_{n-1}^{c}\\ \vdots & \ddots & \vdots \\ x_{n-\alpha }^{1} & \cdots & x_{n-1}^{1} \end{array}\right].\;$$

In the external feedback of the reconstruction data method, as shown in Figure 3b, the $y_{t}$ channel is used simultaneously for the input matrix and output vectors. The input dataset $X_{n}^{c}$ for predicting the n-th element $y_{n}$ of the $y_{t}$ channel consists of the following matrix of size ((c+1)×α).

(3) $$X_{n}^{c}=\left[\begin{array}{c} \begin{array}{ccc} y_{n-\alpha } & \cdots & y_{n-1}\\ x_{n-\alpha }^{c} & \cdots & x_{n-1}^{c}\\ \vdots & \ddots & \vdots \end{array}\\ \begin{array}{ccc} x_{n-\alpha }^{1} & \cdots & x_{n-1}^{1} \end{array} \end{array}\right].\;$$

The model learned through repeated training is used as a reconstruction model at the point in time when data loss occurred. The reconstructed data of the loss channel are fed back to the input data matrix at the next time step. 

## 4. Experimental Verification

### 4.1. Vibration Experiment with Multi-Story Shear Building Model

**Figure 4:**
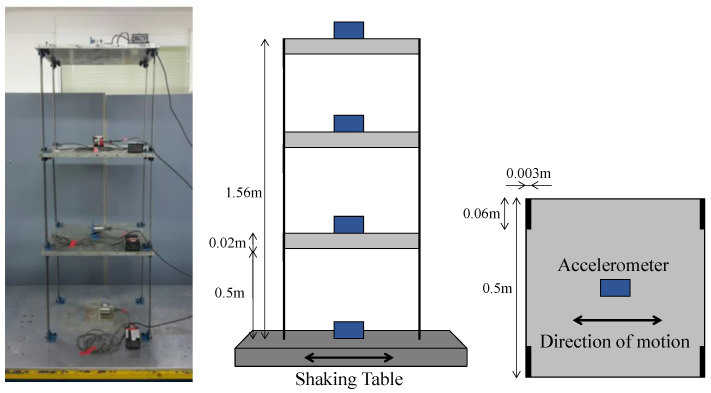
Experimental setup for acquisition of vibration dataset of structure.

To verify the experimental performance of the real-time feedback of the proposed method for reconstruction of data, a series of vibration experiments was conducted to collect the dynamic response data of a multi-DOF structure. The test structure was a three-story single-bay frame, with a total height of 45 cm and a width of 16 cm (Figure 4). It was assembled with two structural elements: a flexible steel plate (50 cm × 3 cm × 4 mm thick) and a rigid aluminum plate (50 cm × 50 cm × 2 cm thick). Aluminum plates were used as floor plates, and both ends of the four steel plates were joined to an L-shaped plate to support each floor plate. Such a prefabricated structure can be expanded to a six-story structure by repeatedly connecting the same structural elements, as explained in Section 4.4.

The structural model was mounted on a uniaxial shake table driven by a mechanical linear actuator, where the rotary motion of an AC servo motor (HC-SFS502, Mitsubishi, Tokyo, Japan) was converted to linear motion. The column connected to the shake table was excited on a plane axis by an analog signal, which is obtained by converting the digital signal generated in MATLAB Simulink using a digital acquisition (DAQ) module, NI-9375 (National Instruments, Austin, TX, USA). Four accelerometers (731A, Wilcoxon, Frederick, MD, USA) with a 100 Hz sampling rate were installed in the center of the shake table and in each floor of the structure to measure the acceleration by the movement of the shake table. White noise and El Centro seismic signals were adopted as the excitations for the shake table to simulate the ambient and seismic motions of the structure. The acceleration measured from the shake table and structure were used to verify the quality of the generated input signals, and to train the RNN model, respectively.

**Figure 5:**
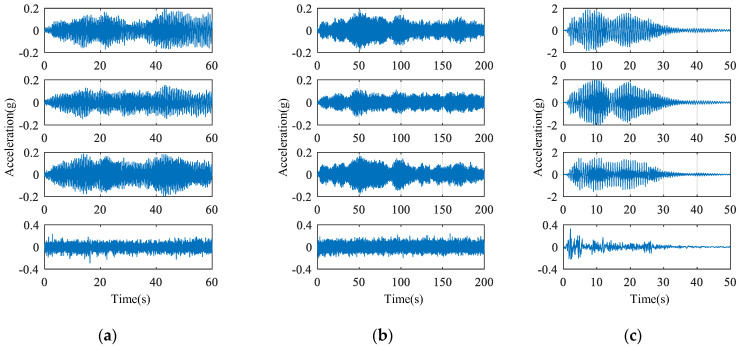
Datasets acquired from building models based on excitation type: the plots provided from top to bottom represent signals of the third, second and first floors, and table, respectively. (**a**) white noise (training and test). (**b**) white noise (validation). (**c**) El Centro (validation).

To build a training dataset for model training, white noise with a frequency range of 0.5 to 30 Hz and a maximum acceleration of approximately 0.3 g was provided as an excitation for 60 s. The behavior of the building, with a maximum acceleration of approximately 0.2 g and a trend of vibration that occurred similarly on the three floors, was used for training and testing for a duration of 40 s and 20 s, respectively (Figure 5a). Two types of validation datasets that were not involved in training were prepared to compare model accuracy: (1) the response of the building to white noise of 200 s duration with a frequency range of 0.5 to 30 Hz and a maximum acceleration of approximately 0.3 g with an acceleration magnitude similar to the dataset used for model generation (Figure 5b); (2) the response of the building excited for 50 s by the El Centro seismic signal with a peak value of 2 g, which is approximately 10-times greater than the maximum acceleration of the dataset used for model training (Figure 5c). In the validation dataset, the sensor on the third floor was assumed to be the lost channel, and its data were reconstructed from the RNN model. 

### 4.2. RNN Model Training and Its Evaluation

Simple RNN, LSTM, and GRU were selected as RNN models for performance comparison of the proposed external feedback of the sensor data reconstruction. The hyperparameters in Table 1 were identical for the three models. The number of input data and hidden layers were set as variables to evaluate the stability of the model generated by the proposed method. The numbers of input data and hidden layers were increased a total of 20 times at intervals of 4, from a minimum of 4 to a maximum of 40, and for a total of 20 times at intervals of 5, from a minimum of 5 to a maximum of 100. The other hyperparameters, such as the optimizer, training loss, learning rate, batch size, and maximum epochs, were fixed.

**Figure 6:**
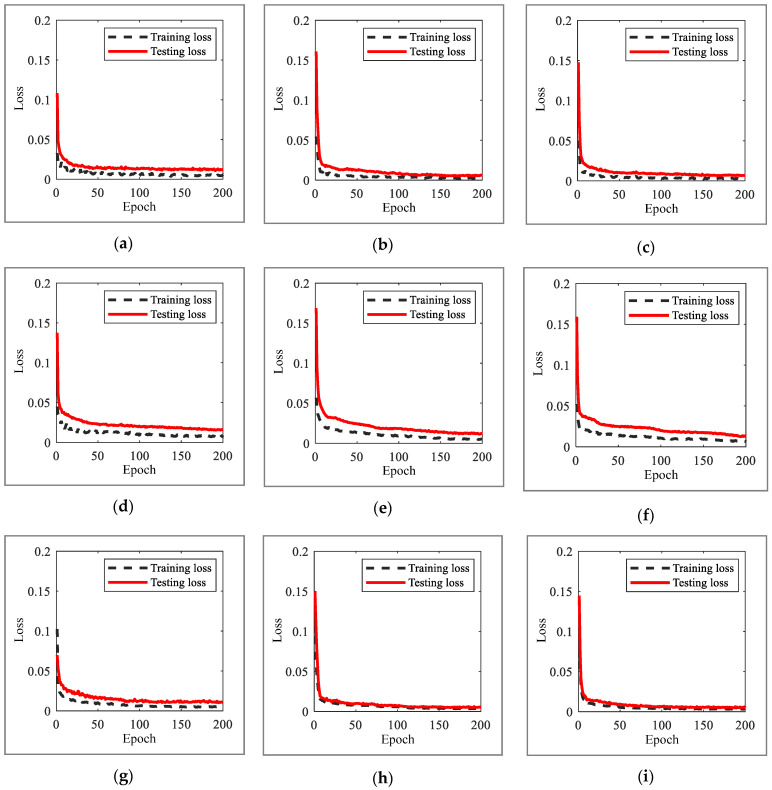

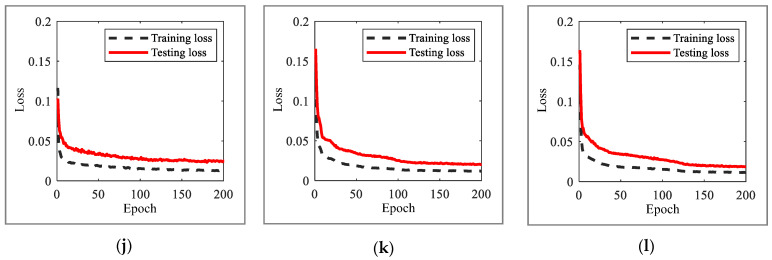
Training and testing the loss in existing and proposed methods based on RNN model and excitation type with respect to the training epochs. (**a**) Simple RNN, white noise (proposed). (**b**) LSTM, white noise (proposed). (**c**) GRU, white noise (proposed). (**d**) Simple RNN, white noise (existing). (**e**) LSTM, white noise (existing). (**f**) GRU, white noise (existing). (**g**) Simple RNN, El Centro (proposed). (**h**) LSTM, El Centro (proposed). (**i**) GRU, El Centro (proposed). (**j**) Simple RNN, El Centro (existing). (**k**) LSTM, El Centro (existing). (**l**) GRU, El Centro (existing).

Model training and evaluation were performed to quantitatively calculate the performance of the external feedback (termed as proposed) and internal feedback inherent in the RNN model (termed as existing). In total, 2400 (12 × 200) models were generated for the number of input vectors and layers in 12 situations, depending on the model type (simple RNN, LSTM, GRU) and the type of excitation (white noise and El Centro seismic signals). Figure 6 shows the training and test losses of the RNN model according to the increase in epochs in the training process of the model and is the average of 200 values of training and test losses acquired in each situation. Overall, there is a rapid loss reduction in less than 10 epochs; subsequently, in the case of the existing method, it gradually decreases to 200 epochs, and in the case of the proposed method, it converges to the minimum loss at approximately 100 epochs. The converged losses of the proposed method were lower than those of the existing method.

### 4.3. Sensor Data Reconstruction from Trained RNN Models

**Figure 7:**
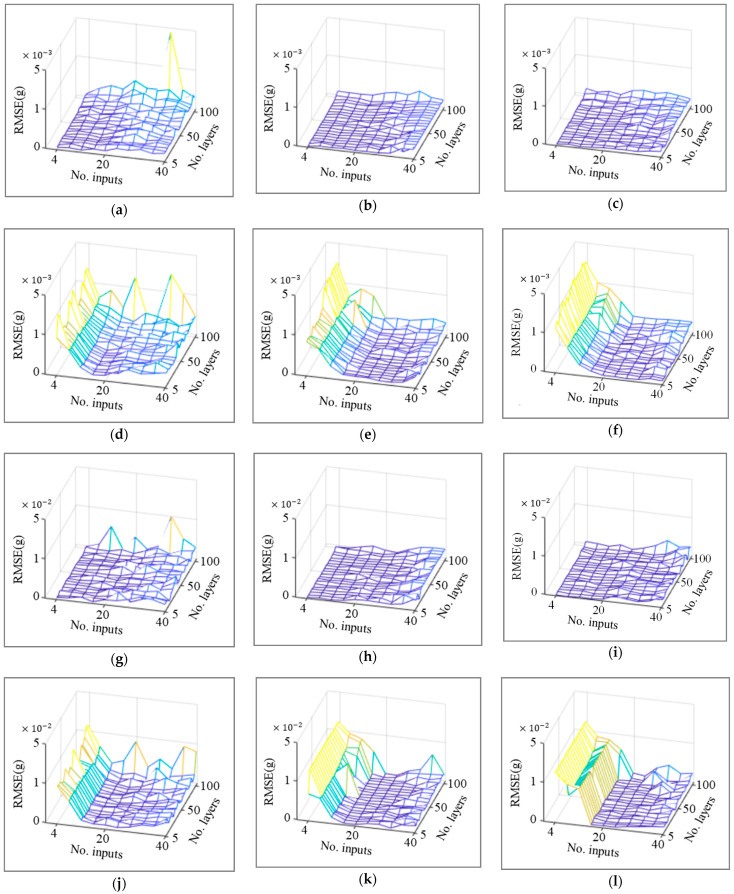
RMSE of existing and proposed methods for different RNN models and excitation types. (**a**) Simple RNN, white noise (proposed). (**b**) LSTM, white noise (proposed). (**c**) GRU, white noise (proposed). (**d**) Simple RNN, white noise (existing). (**e**) LSTM, white noise (existing). (**f**) GRU, white noise (existing). (**g**) Simple RNN, El Centro (proposed). (**h**) LSTM, El Centro (proposed). (**i**) GRU, El Centro (proposed). (**j**) Simple RNN, El Centro (existing). (**k**) LSTM, El Centro (existing). (**l**) GRU, El Centro (existing).

The validation dataset that is not used for training and testing was input to the previously generated 2400 models, and the third-floor data that were assumed as a lost channel were reconstructed. The root mean square error (RMSE, $\sqrt{\underset{k}{\sum }{\left(y_{k}^{ptredicted}-y_{k}^{measured}\right)}^{2}/number\;of\;data\;points}$) between the reconstructed data and measured data is presented as 3D mesh plots in Figure 7. In the case of the existing method, starting from high values, the RMSEs tended to decrease up to 20 input vectors, but gradually increased after 20 input vectors. In addition, unstable results were obtained when the number of layers was close to 100. On the other hand, in the case of the proposed method, low RMSEs are obtained regardless of the number of inputs, which confirms a stable trend of RMSEs, even though RMSEs slightly increase after 20 input vectors. In addition, robust and low RMSEs were obtained for all models, except for the high number of hidden layers of the simple RNN with a simple layer structure. There were no differences according to the type of excitation signal used.

The dynamic response features of the structure can be effectively contained as the length of the input vector increases. The lengthened input vector increases the number of parameters inside the RNN model; therefore, the computation becomes more expensive. Thus, a tradeoff occurs in the determination of the input vector length. In the case of loss data reconstruction by correlation analysis of sensor data, it is confirmed that the dynamic response characteristics are related to temporal correlation, and optimization of the input vector length considering the tradeoff is required in the existing internal feedback. In contrast, the proposed external feedback method of feeding the reconstruction output of the lost channel back to the input dataset reduces the influence of temporal correlation by emphasizing the spatial correlation in the input data between sensor channels. In addition, it was verified that the proposed method can make the hyperparameter tuning process robust, even at a high number of layers.

Table 2 shows the number of input vectors and the RMSE of the models generating the least RMSE through hyperparameter optimization in each mesh. Because the difference in accuracy based on the number of layers was insignificant, it was fixed at 50. The number of input vectors of the existing method according to white noise and El Centro seismic signal is 18.7 and 25.3, respectively. However, in the case of the proposed method, it was reduced to 5.3 and 4, respectively. In addition, the model accuracy evaluated by the RMSEs was reduced to 3.107 $\times \;10^{-3}$ g and 7.277 $\times \;10^{-2}$ g for the proposed method, against 8.620 $\times \;10^{-3}$ g and 20.316 $\times \;10^{-2}$ g in the existing method. It is demonstrated that the RNN model for sensor data reconstruction conducted using the proposed method improves the dependency of the hyperparameter setting and accuracy.

**Figure 8:**
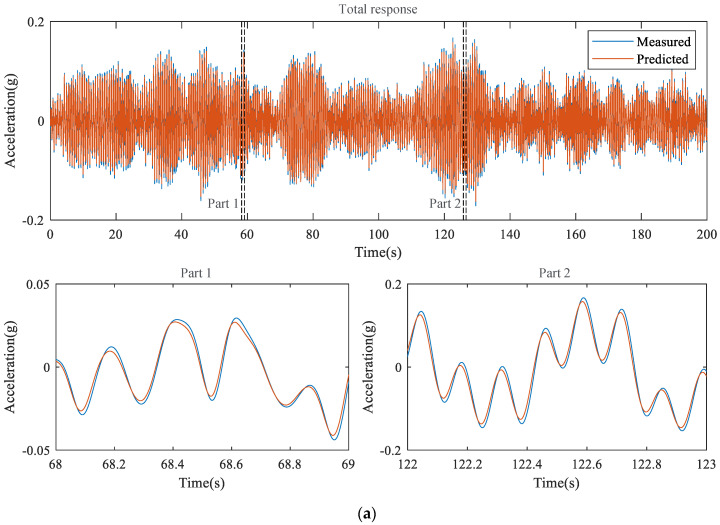

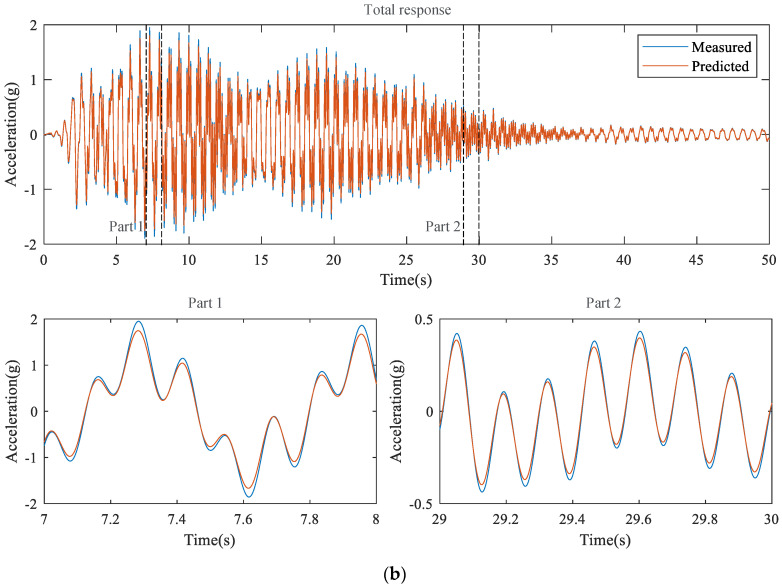
Acceleration time history of the measured and reconstructed data. (**a**) structural vibration due to white noise; (**b**) structural vibration caused by the El Centro seismic signal.

The responses of the structure reconstructed from the proposed external feedback method are compared with those of the measurement in Figure 8, where the model accuracies are 2.147 $\times \;10^{-3}$ and 4.878 $\times \;10^{-2}$, respectively, for the white noise and El Centro seismic signal cases shown in Table 2.

### 4.4. Extended Six-Story Structure Model

**Figure 9:**
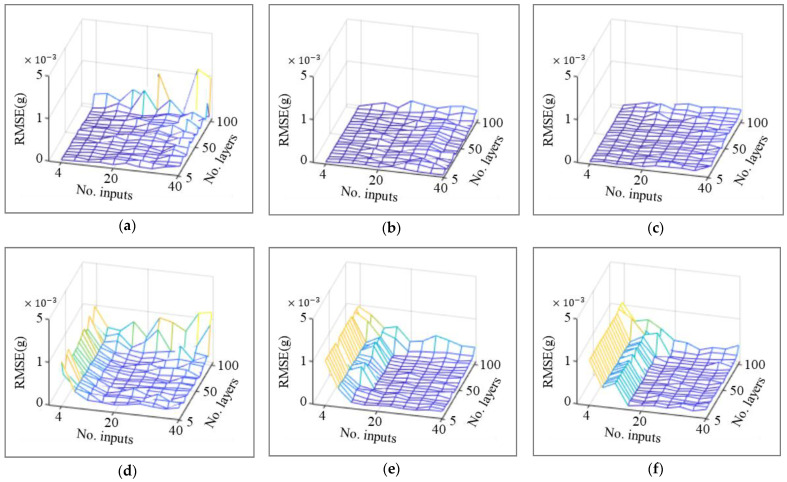
RMSE of existing and proposed methods for different RNN models. (**a**) Simple RNN (proposed). (**b**) LSTM (proposed). (**c**) GRU (proposed). (**d**) Simple RNN (existing). (**e**) LSTM (existing). (**f**) GRU (existing).

To evaluate the effect of the complexity of the multi-DOF structure system on the proposed method, the same mass and stiffness system was extended to the six-story structure model and sensor channel. A three-story prefabricated structure model composed of a steel plate, aluminum plate, and L-shaped plate was further assembled with members of the same size and expanded to six stories. The host channel was assumed to be the sixth floor, and white noise was excited on the shake table. The other experimental conditions remained the same as that for the previous testing structure. As a result of the training, 1200 (2 × 3 × 200) models were generated according to the existing and proposed methods, model types, number of input vectors, and hidden layers. The lost channel of the 200 s validation dataset that was not used for training and testing was reconstructed. The RMSE of the accuracy of the model is presented in the 3D mesh plots in Figure 9. The overall trend was similar to that of the three-story structure model: in the case of the existing method, a high RMSE occurred when the number of vectors was low and decreased sharply to 16 of the input vectors, and in the case of the proposed method, a low RMSE occurred, regardless of the number of input vectors. Model instability at a high number of layers is found in all models of the existing method and in the simple RNN of the proposed model, but the LSTM and GRU of the proposed method resulted in a low and stable RMSE in all models. The optimized models of the existing and proposed methods were derived from LSTM. The number of input vectors and hidden layers and the RMSE are 28, 32, and 7.319 $\times \;10^{-3}$ g, respectively, for the existing method and 4, 24, and 2.871 $\times \;10^{-3}$ g, respectively, for the proposed method. That is, the number of input vectors is reduced by more than six times, and the RMSE by more than two times. Thus, it is proven that the proposed method can generate a stable and high-accuracy RNN model, even when the complexity of the structure increases.

**Figure 10:**
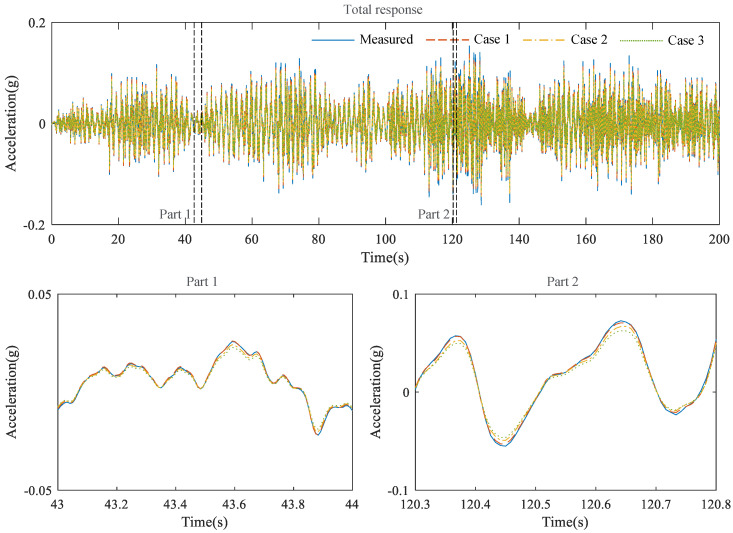
Acceleration time history of reconstructed data of the sixth floor.

The performance of the proposed external feedback method using LSTM was further evaluated under the conditions of multiple sensor channel losses: the sixth-floor sensor loss (Case 1), the fifth- and sixth-floor sensor losses (Case 2); the fourth-, fifth-, and sixth- floor sensor losses (Case 3). In comparison experiments on the previous six-story structure model involving three reconstruction models, data commonly generated at the sixth floor were compared and are depicted in Figure 10 and the quantitative information is tabulated in Table 3. Figure 10 shows the time histories of the measured and reconstructed data acquired in Cases 1 to 3. In general, the measured data and the three reconstructed data were similar. In the zoomed plots, the reconstructed signals tended to be underestimated as the number of lost sensors increased.

## 5. Conclusions

In this study, a real-time external feedback loop was proposed for the use of RNN models, and its effect was quantitatively evaluated through a series of experiments. The proposed RNN model with external feedback was demonstrated and verified with the vibration response dataset obtained from experiments with white noise and El Centro seismic signal excitation in a three-story structure model, and the experiment was extended to a six-story structure model. It was proven that the method proposed simplifies hyperparameter tuning and generates a more accurate model for the RNN-based reconstruction of lost data.

The accuracy of the RNN model was compared using the RMSE between the reconstructed and measured data. Based on the results in the case study, the following conclusions are drawn: the proposed method through the three-story structure model experiment generated a model with robust accuracy, regardless of the number of input data and layers in simple RNN, LSTM, and GRU models. Compared to the use of the conventional RNN models, the number of input data was reduced by four-times, and the RMSEs were reduced by three-times using the proposed external feedback RNN models. In the six-story structure model experiment, under scenarios in which the number of fault sensors was increased up to three channels, robust models with high accuracy were evaluated. For the reconstructed signals on the sixth floor, trivial differences between each reconstruction of the three fault scenarios were confirmed.

## Figures and Tables

**Table 1:** Hyperparameters set in the RNN models.

Hyperparameter	Value
Number of input data (min./max./interval)	4/40/4
Number of hidden layers (min./max./interval)	5/100/5
Optimizer	Adam
Training loss	MAE
Learning rate	0.001
Batch size	72
Maximum epochs	200

**Table 2:** Number of input vectors (NIVs) and RMSE of the optimized RNN models.

			Simple RNN	LSTM	GRU	Mean
White noise	Proposed	NIV	4	4	8	5.3
		RMSE (g)	4.294 $\times \;10^{-3}$	2.147 $\times \;10^{-3}$	2.881 $\times \;10^{-3}$	3.107 $\times \;10^{-3}$
	Existing	NIV	20	16	20	18.7
		RMSE (g)	11.495 $\times \;10^{-3}$	5.256 $\times \;10^{-3}$	9.109 $\times \;10^{-3}$	8.620 $\times \;10^{-3}$
El Centro seismic signal	Proposed	NIV	4	4	4	4
		RMSE (g)	11.037 $\times \;10^{-2}$	4.878 $\times \;10^{-2}$	5.916 $\times \;10^{-2}$	7.277 $\times \;10^{-2}$
	Existing	NIV	16	28	32	25.3
		RMSE (g)	27.910 $\times \;10^{-2}$	15.373 $\times \;10^{-2}$	17.666 $\times \;10^{-2}$	20.316 $\times \;10^{-2}$

**Table 3:** Quantitative information related to reconstructed data.

	Measured Value	Case 1	Case 2	Case 3
Peak (g)	0.1609	0.1417	0.1446	0.1343
RMS (g)	0.0388	0.0377	0.0354	0.0333
RMSE (g)	-	2.781 × 10^−3^	5.751 × 10^−3^	8.815 × 10^−3^

## Data Availability

The data presented in this study are available on request from the corresponding author.
